# A Method for Optimized Monitoring of Indoor Air Quality in Public Buildings

**DOI:** 10.3390/s26144559

**Published:** 2026-07-18

**Authors:** Filippo Ruffa, Grazia Iadarola, Alberto De Capua, Claudio De Capua

**Affiliations:** 1 DIIES—Department of Information Engineering, Infrastructure and Sustainable Energy, Mediterranea University of Reggio Calabria, 89122 Reggio Calabria, Italy; g.iadarola@staff.univpm.it (G.I.); decapua@unirc.it (C.D.C.); 2 dAeD—Department of Architecture and Design, Mediterranea University of Reggio Calabria, 89122 Reggio Calabria, Italy; adecapua@unirc.it

**Keywords:** indoor air quality (IAQ), air quality measurement, air pollutants, thermal comfort, individual exposure, human perception, environmental monitoring systems

## Abstract

A huge effort has been directed towards research and development of new measurement systems for maximizing comfort and safety in public buildings by monitoring indoor air quality (IAQ). In fact, according to World Health Organization, exposure to chemical, biological, and physical agents in poorly ventilated spaces can lead to psycho-physical discomfort as well as respiratory and neurological diseases. Recent advances in the Internet of Things (IoT) have paved the ground for the design and implementation of distributed measurement systems with higher sensor density and computational capacity. While these systems provide accurate assessments of individual rooms, they do not account for personal exposure to varying air quality levels over time. In public buildings such as schools, universities, and workplaces, occupants frequently move between rooms according to predefined schedules, resulting in heterogeneous exposure patterns. To address this issue, this paper proposes an innovative IAQ measurement technique for public buildings, shifting the focus from room-based assessment to occupant-centered assessment. Unlike wearable or portable personal monitors, the proposed technique infers occupant location from the institutional timetable and combines it with the fixed sensor infrastructure already installed in the rooms, requiring no additional devices to be worn. Individual conditions are quantified through a new personalized metric that integrates instantaneous air quality, cumulative individual exposure over time, and thermal comfort into a single index that is evaluated against occupant-specific thresholds. The technique is validated using real-world data, demonstrating higher potential to ensure safety and comfort compared to the state of the art.

## 1. Introduction

In recent years, increasing attention has been directed to the impact of indoor air quality (IAQ) on human health and well-being, especially in public buildings where individuals spend extended periods together, such as schools, elder care facilities, and hospitals [1,2,3,4]. According to the World Health Organization, low IAQ can lead to sick building syndrome (SBS), which commonly affects people who are subject to prolonged exposure to chemical, biological, and physical agents in public buildings with bad ventilation [5]. In fact, IAQ can be significantly worse than outdoor air quality, with levels of pollutants that may be two to five times higher and occasionally more than 100 times higher than in the outdoor case, as highlighted by the United States Environmental Protection Agency [6]. Therefore, people who spend long periods in enclosed spaces with limited air exchange are more likely to experience psycho-physical discomfort. Similar conditions over time can contribute to serious health problems, mainly respiratory diseases but also cardiovascular and mental disorders, and in some cases even cancer [7,8].

Other two factors playing an important role in perceived comfort and health are temperature and relative humidity (RH), which indicates the amount of moisture in the air. For example, a correlation between elevated indoor temperature and higher incidence of symptoms associated with SBS is known in the literature. In fact, maintaining the temperature within an optimal range has been shown to support better performance in workplaces. Occupants tend to be more productive when thermal conditions are stable, generally around values between 21 °C and 25 °C [9]. RH instead affects how heat is perceived and tolerated; when humidity is too low, it can cause dryness and irritation of mucous membranes and respiratory tract, which become more susceptible to infections caused by airborne viruses [10,11]. On the other hand, excessive humidity can promote the survival of some non-lipid enveloped viruses and certain bacteria [12], as well as the growth of mold and dust mites [13,14], making the environment less healthy and potentially contributing to respiratory problems. For this reason, maintaining indoor RH within the 40–60% range is widely considered beneficial for health [15]. Given this premise, it becomes evident that effective environmental monitoring and regulation are vital tools in ensuring healthier conditions. These concerns are particularly critical in the presence of vulnerable populations such as children and elderly, who are more susceptible to the adverse effects of pollutants and inadequate ventilation [16,17,18,19]. In the case of schools, maintaining good IAQ is also essential to learning outcomes [20].

The growing availability of Internet of Things (IoT) technologies has paved the way for new strategies to monitor indoor environments with energy-saving strategies [21,22,23,24,25]. Smart sensors, including low-cost sensors [26], can continuously collect data on key parameters that serve as indicators, enabling proper IAQ assessment as well as more effective management of ventilation systems [27,28]. The hardware employed for data acquisition can consist of high-cost real-time equipment such as NI RIO [29], but there are also several types of low-cost systems based on general-purpose microcontroller units such as Arduino [30,31,32,33,34,35,36], raspberry-PI [37,38], and ESP8266 [39,40]. The most common communication and data transfer technologies in IoT are WiFi [29,31,32,37,39,41], Bluetooth [34,35,39,42], and ethernet [43].

In research on green building schemes and standards, the focus is monitoring temperature and humidity [29,37,41,42,44,45] along with three commonly studied air pollutants, namely, carbon dioxide (CO_2_), total volatile organic compounds (TVOCs), and particulate matter [3,27,30,46,47,48,49,50,51,52]. In a recent work, an innovative strategy for IAQ monitoring systems was proposed including real-time data processing to obtain appropriate indices, which not only considers both the instantaneous concentration of single pollutants and the duration of exposure [53]. The system proposed by [53] considers user needs as well as pollutant safety thresholds set by current regulations; when necessary, ventilation systems are activated to restore air quality levels. Moreover, by carefully adjusting indoor ventilation, CO_2_ levels [54,55] and concentration of particulate matter (PM) can be decreased while maintaining constant humidity [56]. The resulting indoor environments are healthier, with markedly reduced potential for disease transmission [57].

The greatest part of existing buildings were devised before modern health and energy regulations, and still lack adequate systems for real-time IAQ monitoring. In this context, the present work proposes a method for optimized IAQ monitoring in public buildings such as educational institutes and workplaces by proposing safety indices personalized for each occupant. In indoor living environments, people often share enclosed spaces and move between rooms according to a time-table; as a result, individuals present in the same environment at a given time may not have been exposed to the same levels of pollutants throughout the day. This heterogeneity in individual exposure has been documented in the literature through different and complementary approaches. From an empirical standpoint, a recent study employing continuous CO_2_ monitoring across multiple classrooms in different schools reported time-above-threshold percentages ranging from 0% to 8.7% of the school day despite comparable building types, regulatory context, and occupant age groups [58]. These results confirm that room-level conditions can vary substantially even within a homogeneous building category. From a methodological standpoint, individual exposure has also been addressed through statistically modeled approaches. Such approaches, including combining smartphone-based location tracking with land-use regression models to estimate pollutant exposure in general urban settings [59] and occupancy detection and tracking technologies for smart buildings, were comprehensively reviewed in [60], including passive infrared, Bluetooth-based localization, and vision-based methods. However, the former target general urban settings rather than structured environments with predictable occupancy schedules, such as schools or public offices, while the latter have primarily been applied to building management objectives such as HVAC control, energy optimization, and security rather than to the construction of individualized pollutant exposure metrics. Taken together, these lines of research confirm the relevance of occupant-centered exposure assessment, but leave a gap for structured and schedule-driven environments where individual exposure must be linked directly to a personalized comfort metric.

The approach proposed in this paper addresses this gap by leveraging the institutional timetables already available in such structured environments to infer occupant location and directly link it to a personalized exposure index, thereby avoiding the need for additional tracking hardware or wearable devices. This is particularly advantageous in contexts involving minors, where cost, privacy, and compliance constraints often limit the feasibility of continuous individual tracking. The solution proposed in this work uses the data acquired by the sensor nodes in the various rooms, then uses the institute time-table to compute a personalized comfort level index for each individual user. This study builds upon and extends the methodological framework proposed in [61], which introduced personalized IAQ assessment. In particular, the present work broadens the analysis by including thermal comfort evaluation and assessing the specific contribution of each environmental component to the overall comfort condition experienced by each occupant. The technique is validated using real-world data [62,63] from a field study at a K-12 private school in the suburbs of Melbourne, Australia, extracted from the PhyisioNet database [64]. The aim of this validation is to demonstrate that standard air quality monitoring alone is not sufficient to ensure adequate safety levels for public buildings such as schools and universities. The results highlight the need for solutions that account for the specific environmental conditions to which each individual is exposed within the institution.

The rest of this paper is structured as follows: Section 2 describes the proposed methodology and introduces a new metric for joint evaluation of air quality, exposure to air quality levels, and thermal comfort; Section 3 describes implementation aspects for a specific case study, i.e., air quality monitoring in educational buildings; Section 4 describes the validation methodology and shows the results obtained; finally, Section 5 reports the conclusions.

## 2. Methodology

The approach presented in this paper relies on the introduction of a new metric, d(ID), which quantifies the distance from comfort thresholds in terms of air quality, exposure to air quality levels, and thermal comfort. These comfort thresholds can be defined individually on the basis of personal needs and health conditions. The index d(ID) is specific to each user identified by a unique ID number inside the building, and relies on three key factors: air quality in the room; individual exposure to air quality levels, accounting for exposure time to pollutants; and thermal comfort. Let these three contributions be defined as follows:

(1) $$d_{IAQ}\left(ID\right)=I_{IAQ}-th_{IAQ}\left(ID\right),$$

(2) $$d_{EIAQ}\left(ID\right)=EI_{IAQ}\left(ID\right)-th_{EIAQ}\left(ID\right),$$

(3) $$d_{TC}\left(ID\right)=I_{TC}-th_{TC}\left(ID\right),$$

where $I_{IAQ}$ is the air quality index of the room, $I_{TC}$ is the thermal comfort index, and $EI_{IAQ}\left(ID\right)$ is the index of exposure to air quality levels for the specific user ID. We calculate $EI_{IAQ}\left(ID\right)$ using measurement data from sensor nodes installed in the various rooms of the institute where the person identified by their exclusive ID number has actually been located during the day. The minimum thresholds for $I_{IAQ}$, $EI_{IAQ}\left(ID\right)$, and $I_{TC}$ are respectively $th_{IAQ}\left(ID\right)$, $th_{EIAQ}\left(ID\right)$, and $th_{TC}\left(ID\right)$, which are defined individually on the basis of health conditions and personal needs for each user identified by their unique ID number.

The d(ID) index is then defined for each individual user as follows:

(4) $$d\left(ID\right)=min(d_{IAQ}\left(ID\right),d_{EIAQ}\left(ID\right),d_{TC}\left(ID\right)).$$

This metric, which evaluates individual exposure over time, complements traditional approaches to monitoring and controlling IAQ that focus exclusively on ensuring the immediate healthiness of living spaces. Section 2.1 and Section 2.2 explain in detail how to obtain the air quality and thermal comfort indices mentioned above.

### 2.1. Air Quality

According to [53], the IAQ index is defined as follows:

(5) $$I_{IAQ}=min\left(I_{CO},I_{CO2},I_{TVOC},I_{PM2.5}\right).$$

The selection of IAQ evaluation parameters was based on a comprehensive review of the current scientific literature along with consideration of relevant regulations across multiple regions [65]. Aligning with green building principles and established standards (see Table 4 in Mujan et al. 2021 [30]), recent studies [3,46,47,48,49,50,51,52] have emphasized the importance of three key air pollutants: carbon dioxide (CO_2_), total volatile organic compounds (TVOCs), and fine particulate matter (PM2.5).

High CO_2_ concentrations can accumulate in enclosed spaces with poor ventilation, potentially reaching levels between 5000 ppm and 6000 ppm. However, maintaining good IAQ requires keeping CO_2_ levels below 1000 ppm [66]. Monitoring CO_2_ serves as a valuable tool for assessing both ventilation effectiveness and building occupancy. By analyzing indoor CO_2_ concentrations, we can gain insights into factors such as the number of occupants and the rate of fresh air exchange [67].

The analysis also includes particulate matter due to potential links between their concentration and the spread of respiratory viruses [68]. PM exposure has been connected to respiratory illnesses such as asthma, infections, cardiovascular issues, and lung cancer [69]. Data provided by the Victoria EPA Institute (Australia) [70] categorizes PM levels based on both hourly and 24-h average concentrations.

Indoor environments can also harbor significant presence of volatile organic compounds (VOCs) [71]. Common household products, combustion sources, air fresheners, and even building materials themselves all contribute to VOC emissions within residences [72]. Exposure to these chemicals can trigger health problems ranging from eye and respiratory irritation to dizziness, nausea, headaches, and allergic reactions. Furthermore, some VOCs are classified as carcinogens [73]. Therefore, monitoring VOC concentration serves as a crucial parameter for assessing overall IAQ [50,74].

Additionally, a recent work [53] suggested including CO levels in IAQ monitoring. CO is an odorless, colorless, and highly toxic gas that can be produced indoors by sources such as unvented kerosene and gas space heaters, leaking chimneys and furnaces, back-draft from furnaces or gas water heaters, and other sources.

The single pollutant indices are defined as follows:

(6) $$I_{CO}=100-58.7\log\frac{C_{CO}}{1.7},$$

(7) $$I_{CO2}=100-64.2\log\frac{C_{CO2}}{415},$$

(8) $$I_{TVOC}=100-82.8\log\frac{C_{TVOC}}{186},$$

(9) $$I_{PM2.5}=100-85\log\frac{C_{PM2.5}}{10},$$

where $C_{CO}$, $C_{CO2}$, $C_{TVOC}$, and $C_{PM2.5}$ are respectively the measured concentrations of CO, CO_2_, TVOC, and PM2.5. The indices are in logarithmic scale and are limited between 0 and 100; the value of the index is 100 if the measured concentration of the pollutant is equal to the ideal concentration [3] and 0 if it reaches the limit defined by regulations for short-time exposure [65].

Furthermore, Ref. [53] proposed a new method for calculating IAQ index which considers both the concentration of single pollutants and the exposure time. The literature clearly establishes that the impact of a toxic substance on human health depends on both its concentration and the duration of exposure. Thus, a concentration that is safe for brief exposure could become toxic if the exposure time increases to an hour or more. For this reason, a new index related to individual users is defined in this paper, representing an Exposure Index (EI):

(10) $$EI_{IAQ}\left(ID\right)=min\left(EI_{CO},EI_{CO2},EI_{TVOC},EI_{PM2.5}\right)$$

where $EI_{CO}$, $EI_{CO2}$, $EI_{TVOC}$, and $EI_{PM2.5}$ are the exposure quality indices of CO, CO_2_, TVOC, and PM2.5, respectively.

The $EI_{IAQ}$ index, as described in [53], estimates IAQ exposure within a room. However, in contexts where people frequently change rooms, such as workplaces or educational institutions, its applicability is limited. For this reason, in this paper we propose an adaptation of the $EI_{IAQ}$ index so that it can measure the exposure to IAQ levels of each individual occupant within the building. This is achieved by tracking user movements inside the building and continuously collecting air quality measurements from the room in which the user is located. Identifying each user with a unique ID number, the $EI_{IAQ}$ index becomes $EI_{IAQ}\left(ID\right)$, as shown in (10).

For ease of reading, a generic pollutant “x” is considered, and the exposure index is obtained as follows:

(11) $$EI_{x}=100-\alpha \left(t\right)\log\frac{\int_{t0}^{t1}C_{X}dt}{\int_{t0}^{t1}C_{X|0}dt}$$

where $C_{X}$ is the measured quantity of pollutant “x”, $C_{X|0}$ is the concentration of the same pollutant in ideal conditions, and α(t) is a function that considers the toxic levels of the pollutant defined by the current regulation for different exposure times, expressed as

(12) $$\alpha \left(t\right)=\alpha_{0}+\alpha_{1}t+\alpha_{2}t^{2}.$$

The physical rationale behind the formulation of α(t) is as follows. For a given pollutant *x*, regulatory standards define maximum permissible concentrations for different exposure durations (e.g., 15-min, 1-h, 8-h, and 24-h thresholds). The coefficient α(t) is determined by imposing $EI_{x}=0$ exactly when the cumulative integral of concentration equals the regulatory permissible exposure limit for the corresponding duration. This yields a set of known $\left(\bar{t},\alpha \right)$ pairs, one for each available regulatory threshold. When standards from different jurisdictions define different limits for the same exposure duration, the most conservative (lowest) permissible concentration is adopted in order to guarantee maximum occupant safety. A quadratic least-squares fitting through these known points then produces the coefficients $\alpha_{0}$, $\alpha_{1}$, and $\alpha_{2}$ in Equation (12).

The pollutant-specific nature of α(t) reflects the substantially different dose–response relationships that characterize each substance. The regulatory sources used to derive these coefficients are reported in Table 1, while Table 2 summarizes the baseline concentrations $C_{X|0}$ considered and the $\alpha_{0}$, $\alpha_{1}$, and $\alpha_{2}$ coefficients obtained for each pollutant.

### 2.2. Thermal Comfort

Thermal comfort is a critical component of indoor environmental quality and is essential for ensuring the well-being and productivity of building occupants [1,9,15]. The thermal comfort index is calculated based on various parameters that influence the perception of comfort. Generally speaking, the calculation and evaluation of temperature increase in buildings is guided by established standards [75,76]. The thermal comfort index aims to predict thermal comfort levels for occupants. This index is influenced by environmental factors such as air temperature (°C), mean radiant temperature (°C), relative humidity (%), and air velocity (m/s) as well as by personal factors such as clothing insulation (Clo) and metabolic rate (Met). All of these factors are integrated into the predicted mean vote (PMV) model, which is widely used to assess thermal comfort [77,78,79,80,81,82,83]. The PMV model, developed by Fanger [84] and standardized in ISO 7730 [85], predicts the mean thermal sensation vote of a large group of people on a seven-point thermal sensation scale ranging from −3 (cold) to +3 (hot). The PMV index is linked to the predicted percentage of dissatisfied (PPD), which quantifies the percentage of people likely to feel thermally uncomfortable.

The thermal comfort index ($I_{TC}$) is derived directly from the PPD, representing the percentage of occupants satisfied with the thermal environment:

(13) $$I_{TC}=100-PPD.$$

This index provides a measure of thermal comfort, where a higher $I_{TC}$ indicates better thermal comfort conditions.

The calculation and assessment of thermal comfort adheres to the following standards and guidelines:

- ISO 7730 (2005): Ergonomics of the Thermal Environment—Analytical Determination and Interpretation of Thermal Comfort Using Calculation of the PMV and PPD Indices and Local Thermal Comfort Criteria [85].
- ASHRAE 55 (2017): Thermal Environmental Conditions for Human Occupancy [86].
- EN 16798-1 (2019): Energy Performance of Buildings—Ventilation for Buildings, Part 1: Indoor Environmental Input Parameters for Design and Assessment of Energy Performance of Buildings Addressing IAQ, Thermal Environment, Lighting, and Acoustics [87].

In practice, most cases only allow for continuous measurement of ambient temperature $T_{a}$ and relative humidity RH in buildings via low-cost sensors. Localized measurements of air velocity $v_{a}$ are often unavailable. The other parameters involved in ISO 7730 model, such as metabolic rate *M* and clothing insulation $I_{cl}$, cannot be directly observed. Without estimating these parameters, the ISO 7730 model cannot be used for real-time measurements in most practical cases.

To address this issue, assumptions were made to reflect the specific context of a public building. Accordingly, key parameters were set to plausible nominal values. Metabolic rate was set to M=1.1 Met, a typical average for sedentary activity in offices or schools. Clothing insulation was set to $I_{cl}=0.5$ Clo for summer, 0.7 Clo for mid-season, and 1.0 Clo for winter, with air velocity $v_{a}=0.10$ m/s. Atmospheric pressure was fixed at 101.3 kPa and external work to W=0 W/m^2^. A reference humidity $RH_{0}$ was fixed to 50% (or alternatively to the seasonal median of the measurements). Assumptions were also made regarding operative temperature ($T_{op}$), which represents a combined measure of air temperature ($T_{a}$) and mean radiant temperature ($T_{r}$). In public buildings such as schools and workplaces where air velocities are typically low (<0.2 m/s), the convective and radiative heat transfer coefficients are comparable [86]; under this assumption, the operative temperature can be assumed to equal the air temperature $T_{op}\approx T_{a}$ provided that the mean radiant temperature is also close to the air temperature, as is typically the case in well-insulated buildings without significant radiant asymmetries.

The neutral operative temperature $T_{op,0}$ was then obtained by solving

(14) $$PMV(T_{op,0},RH_{0};M,I_{cl},v_{a})=0.$$

This defines the neutral point $\left(T_{op,0},RH_{0}\right)$ around which comfort variations are evaluated.

To express PMV variation as a function of only $T_{op}$ and RH, we obtained local sensitivities $s_{T}$ and $s_{RH}$ using centered finite differences. The other parameters involved in the ISO 7730 model were varied within a realistic context-related domain to consider the worst-case scenario.

With only temperature and humidity sensor data, the PMV deviation from the neutral point can be expressed as

(15) $$\Delta PMV\leq s_{T}\;|T_{op}-T_{op,0}|\;+\;s_{RH}\;\left|RH-RH_{0}\right|.$$

Finally, the percentage of dissatisfied occupants (PPD) is estimated through the following standard relation:

(16) $$PPD=100-95\exp\left(-0.03{\left(\Delta PMV\right)}^{4}-0.22{\left(\Delta PMV\right)}^{2}\right).$$

This worst-case approach guarantees that the resulting ΔPMV bound is always a conservative upper estimate. By selecting the maximum sensitivity coefficients $s_{T}$ and $s_{RH}$ across the full range of plausible *M*, $I_{cl}$, and $v_{a}$ values, the computed ΔPMV can only overestimate the true deviation from the neutral point. Consequently, $I_{TC}$ derived from Equation (13) underestimates the fraction of thermally satisfied occupants rather than overestimating it, which is the conservative direction appropriate for a safety-oriented application. A variation of ±0.1 Met in *M* corresponds to a change of approximately ±0.03 PMV units under the considered conditions, while a variation of ±0.1 Clo in $I_{cl}$ corresponds to approximately ±0.02 PMV units; both are subsumed within the worst-case sensitivity coefficients reported in Equation (15).

## 3. Case Study: Air Quality Monitoring in Educational Buildings

A specific application of interest where the proposed methodology proves particularly useful in ensuring individual safety and comfort is the IAQ monitoring in educational buildings such as schools and universities, where users often change rooms at scheduled times. In such contexts, simple IAQ monitoring in rooms is not sufficient to assess the exposure of each person to toxic substances, and an individual approach is preferable. The methodology described in Section 2 requires the implementation of a distributed measurement system which consists of a number *n* of sensor nodes for the detection of IAQ levels and thermal comfort. The sensor nodes include modules for the detection of temperature, humidity, and the concentration of some pollutants in the air. Identification of the substances involved in assessing IAQ is the result of accurate study and synthesis of both the state of the art and the current regulations, as described in Section 2, and focuses on four pollutants that are highly representative of air quality conditions in enclosed spaces: CO, CO_2_, TVOC, and PM2.5. Each sensor node includes modules for detecting the concentration of the above-cited pollutants in air together with connection modules for IoT implementation. The number of sensor nodes is m>k, where *k* is the number of monitored classrooms, since it is necessary to have at least one sensor node for each classroom.

Some regulations and standards define a minimum number of sensor nodes for a defined area; for instance, WELL recommends a minimum of one sensor node per 325 m^2^ of area for spaces < 3250 m^2^, while RESET recommends a minimum of one sensor node per 500 m^2^ of area. However, some studies in the literature have suggested that temperature, humidity, and particulate matter are not uniformly distributed in a room within the three spatial dimensions [88,89,90]; thus, in large rooms such as grand halls and gyms it is advisable to have more than one sensor node for accurate measurement. The sensor nodes should be uniformly distributed, considering the type of room and density of people, and should be placed at breathing height, typically 1.1 to 1.7 m above the ground.

The sensor nodes collect and send measurement data through the internet to an interactive platform running on a remote server. The platform assigns a unique identifier to each sensor node and collects the received data in 24-h packets, which are saved in files with progressive numbering according to the format SENSOR_ID_YEAR_MONTH_DAY.

To enable personalized exposure assessment and improve the spatial and temporal resolution of air quality monitoring, the data processing system integrates a scheduling file that contains the institutional timetable. This file provides detailed information on user occupancy and classroom transitions throughout the day, allowing for dynamic association of individuals with the specific indoor environment they occupy at any given time.

Each user, whether student, teacher, or administrative staff, is assigned a unique identifier (User ID), which is used to compute the d(ID) index. This index is derived by correlating the user’s presence as defined by the timetable with the air quality and thermal comfort data recorded in the corresponding classroom during each time slot. The computation methodology for d(ID) is detailed in Section 2, and its expression is given in Equation (4).

**Figure 1:**
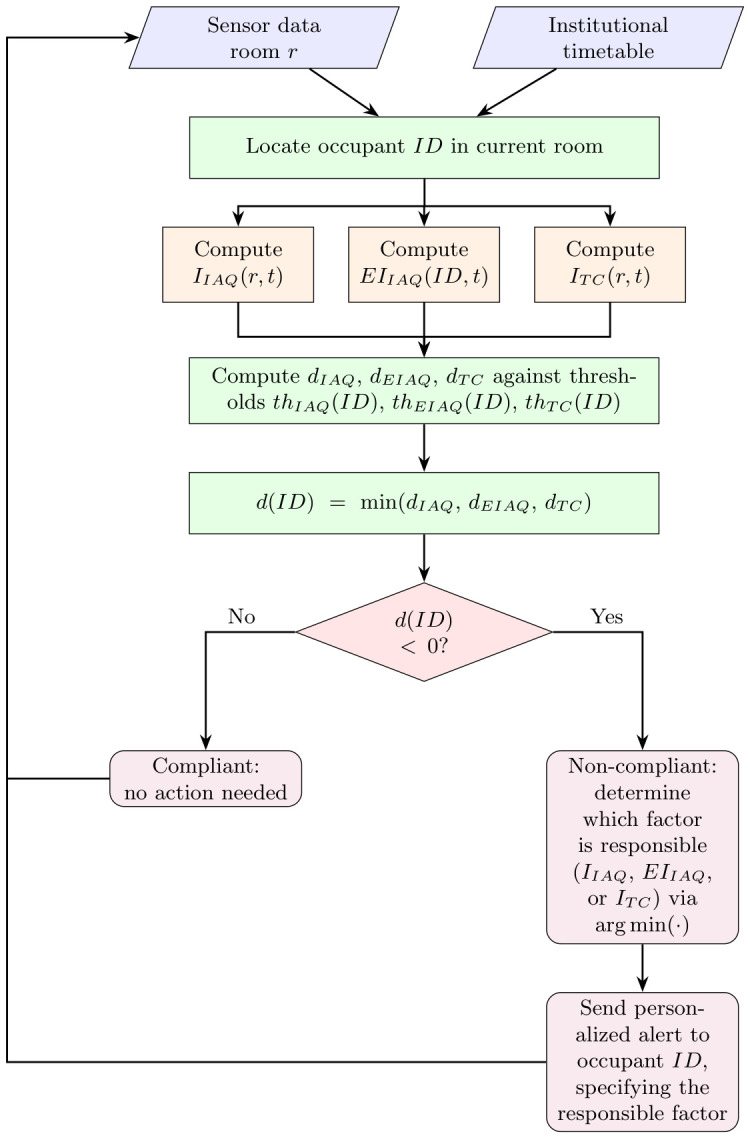
Computational pipeline for generation of the personalized index d(ID), from sensor and timetable acquisition to personalized feedback.

Real-time safety checks can be performed in each classroom, i.e., if one or more d(ID) values associated with users currently present in a given room falls below zero, corrective action can be undertaken to restore acceptable conditions. The definition and optimization of such corrective actions, for instance through the control of the ventilation system, is beyond the scope of the present work, which focuses on the monitoring stage and represents a natural direction for future developments. The system can also provide personalized feedback so that each user can be informed about their personal d(ID) in real time. The complete flow of operations, from sensor and timetable acquisition to the computation of d(ID) and the identification of the responsible factor in case of non-compliance, is summarized in Figure 1.

### Validation on Real Measurements

This section serves to consider the effectiveness of the proposed methodology on real measurements. For this purpose, data from a field study at a K-12 private school in the suburbs of Melbourne, Australia have been used [62,63]. The datasets, extracted from the PhyisioNet database [64], contain two elements:

- In-Gauge dataset: This dataset comprises information from a 5-month longitudinal field study. It includes data from two outdoor weather stations, indoor weather stations across 17 classrooms, and temperature sensors on occupant-controlled room air conditioner vents. The data for each classroom are organized into individual datasets, logged every 5 min.
- En-Gage dataset: This 4-week cross-sectional study monitored 23 students and 6 teachers distributed in the classrooms according to a timetable.

In particular, the aim is to demonstrate that the proposed approach guarantees higher standards of safety in buildings by adding a further level of monitoring and control tailored to each individual. The data in this case study, which is representative of common conditions in educational buildings such as schools and universities, are employed to demonstrate that even when air quality levels are considered acceptable, they may still be insufficient to guarantee adequate safety levels for each occupant when considering exposure throughout the day. Restricting our focus to this goal, only data segments that include information on the presence and distribution of each of the 23 study participants within the classrooms are considered. The instrumentation used by Gao et al. [63] for IAQ monitoring consisted of Netatmo healthy home coach systems, the specifications of which are reported in Table 3.

As can be seen in Table 3, the In-Gauge dataset includes temperature, humidity, and CO_2_ measurements from the Netamo healthy home coach stations. The algorithm described in Section 2 considers three further pollutants, i.e., TVOC, CO and PM2.5.However, as shown in Equations (5) and (10), both $I_{IAQ}$ and $EI_{IAQ}\left(ID\right)$ are obtained as the minimum value between the corresponding indices referring to single pollutants; thus, lack of data relating to other pollutants does not prevent us from providing an assessment of air quality based only on the available data. Specifically, CO_2_-only validation provides a conservative lower-bound estimate of actual IAQ conditions; hence, the minimum operator in Equations (5) and (10) implies that including TVOC, CO and PM2.5 data could only maintain or lower the index values, never raise them.

## 4. Results

In this Section the results obtained by applying the methodology on the case study are presented. The dataset is analyzed and the corrupted portions are eliminated: the monitoring of classroom environmental parameters and the simultaneous information on the presence and distribution of the students is available only for 12 days, from 2 September 2019 to 14 September 2019, involving a total of 23 individuals (15–17 years old, 13 female and 10 male). The distribution of students by classroom and group can be found in [63].

### 4.1. Analytical Methodology

Air quality data were aggregated per participant and per day according to the distribution of students across classrooms. For each participant and each day, these data were used to compute $I_{IAQ}$ using Equations (5) and (7), $EI_{IAQ}\left(ID\right)$ using Equations (10) and (11), and $I_{TC}$ using Equations (13), (15) and (16).

**Figure 2:**
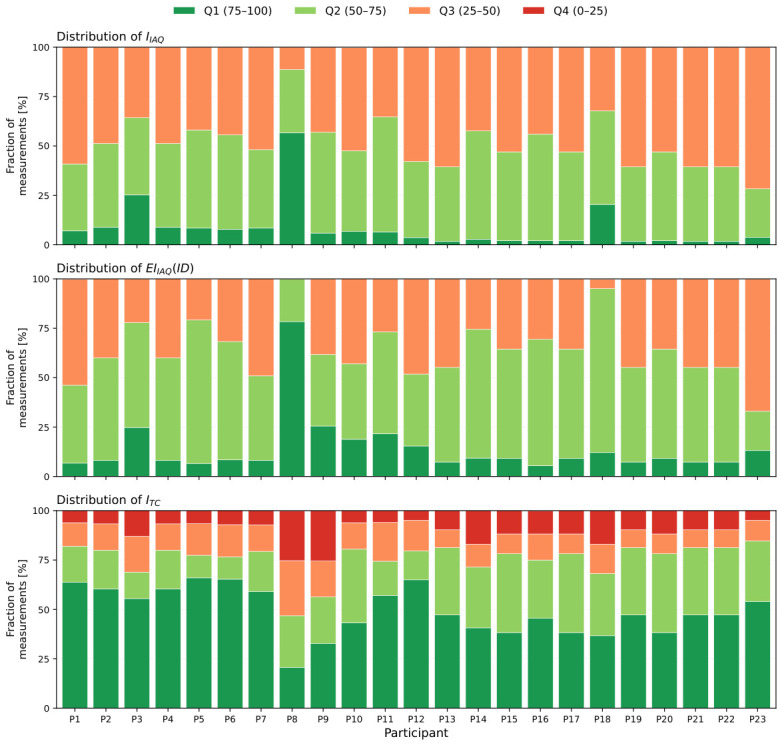
Per-participant distribution across quartiles (Q1–Q4) of the three indices $I_{IAQ}$, $EI_{IAQ}\left(ID\right)$, and $I_{TC}$.

After obtaining the individual indices for each participant and each day, two complementary analyses were performed: the first used the Pearson correlation to quantify how strongly the indices relate to each other, while the second focused on identifying the distribution of the calculated indices. To this end, the range of each index (0–100) was divided into quartiles: Q1 (75–100), Q2 (50–75), Q3 (25–50), and Q4 (0–25). Comparing the distribution of values across quartiles highlights those cases in which one index occupied the upper quartiles (Q1–Q2) while another simultaneously fell within the lower quartiles (Q3–Q4). These discrepancies are particularly relevant because they show that relying on a single index is not sufficient to capture the full IAQ complexity. Finally, the d(ID) index was computed for each participant and for each day, then compared with the state of the art to assess how effectively each method detects non-compliance conditions. Processing the data for each participant and dividing the range of indices into quartiles, no values were found in Q4 for $I_{IAQ}$ and $EI_{IAQ}\left(ID\right)$ out of a total of 6404 analyzed points for all participants and over the entire duration of the monitoring. This means that CO_2_ values never exceeded the levels imposed by the regulations, and that the respective quality indices never fell below 25%. In the remaining quartiles, the data were divided as follows: regarding $I_{IAQ}$, 50.3% fell in Q3, 42.5% in Q2, and 7.1% in Q1; regarding $EI_{IAQ}\left(ID\right)$, 37.0% fell in Q3, 52.0% in Q2, and 11.0% in Q1, with differences between individuals, as shown in Figure 2 for $I_{IAQ}$ and $EI_{IAQ}\left(ID\right)$.

The correlation degree between the two indices is strong (R = 0.72, *p* < 0.001), indicating that they tend to vary in a similar manner across the dataset, which is consistent with the fact that both indices are based on the same CO_2_ measurements.

**Figure 3:**
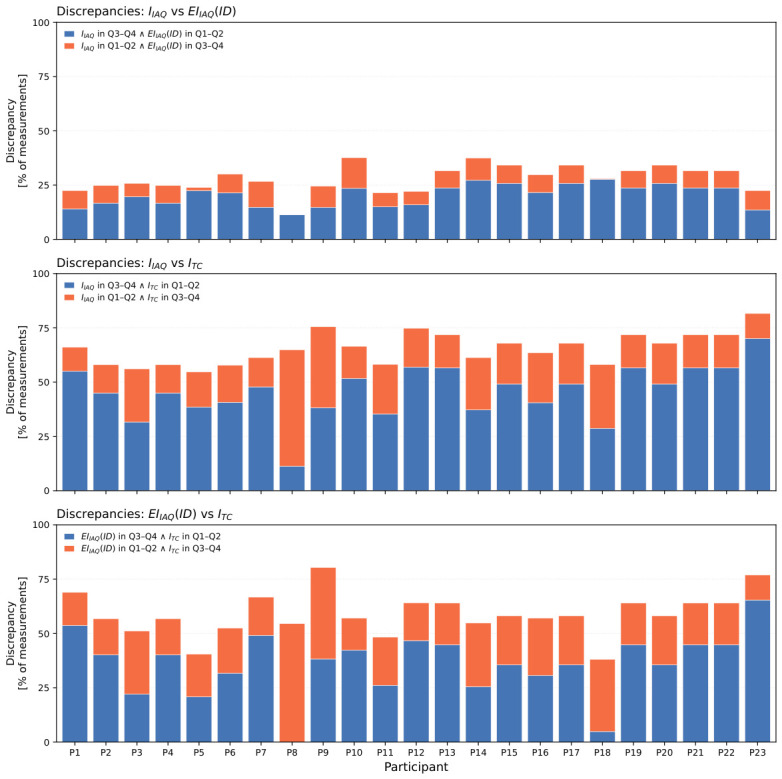
Per-participant discrepancies between pairs of indices. For each pair, the two directional contributions are stacked, so that the total bar height corresponds to the total discrepancy.

**Figure 4:**
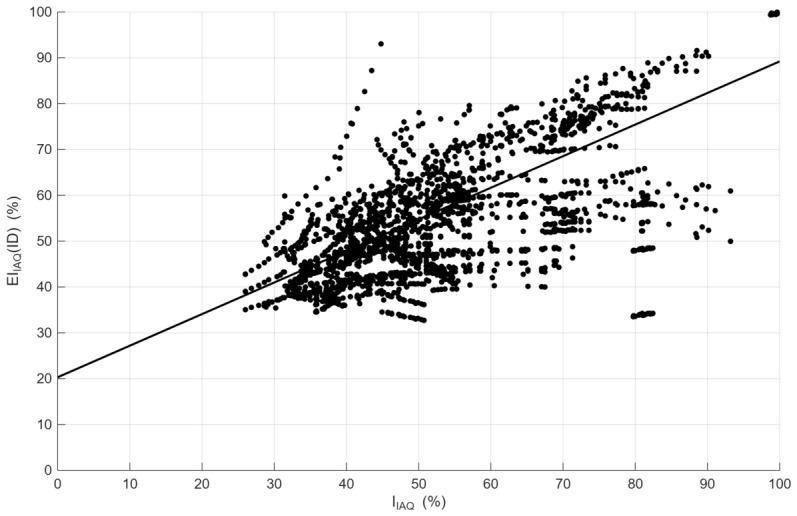
Scatter plot of $EI_{IAQ}\left(ID\right)$ and $I_{IAQ}$ around the fitted regression line.

However, as summarized in Figure 3, particularly interesting is that the two indices diverge in a consistent part of the dataset. This can also be seen in Figure 4, which shows the distribution of points around the regression line. In detail, $EI_{IAQ}\left(ID\right)$ falls within the upper quartiles (Q1–Q2) for 21.0% of the data, indicating exposure to good air quality levels, while $I_{IAQ}$ falls within the lower quartiles (Q3–Q4), indicating non-optimal air quality of the room. In 7.6% of the data, $I_{IAQ}$ falls in Q1 or Q2, with $EI_{IAQ}\left(ID\right)$ in Q3 or Q4. Considering both conditions, the total percentage of discrepancies between the two indices is equal to 28.6%. Figure 3 reports the details of the discrepancies for each participant in the study.

In summary, the data confirm the possibility of this condition, supporting the need to monitor both indices in order to ensure higher safety standards.

Next, thermal comfort was evaluated by computing the $I_{TC}$ index using Equations (13), (15) and (16). Prior to data processing, it was necessary to determine the neutral operative temperature $T_{op,0}$ using Equation (14) along with sensitivity coefficients $s_{T}$ and $s_{RH}$. The dataset used for this validation included measurements collected in a school in Australia between 2 September 2019 and 14 September 2019, corresponding to the winter season. Therefore, to calculate the neutral point $T_{op,0}$, the following assumptions were made: $RH_{0}=50\%$, M=1.1 Met, $I_{cl}=1.0$ Clo, $v_{a}=0.1$ m/s. To calculate the sensitivity coefficients $s_{T}$ and $s_{RH}$, a worst-case analysis was performed by varying the parameters M∈[1.0,1.2] Met, $I_{cl}\in \left[0.8\text{–}1.0\right]$ Clo, and $v_{a}\in \left(0.05\text{–}0.2\right)$ m/s. As a result, the following formulations were obtained.

(17) $$T_{op,0}=22.7\;{}^{\circ}C$$

(18) $$\left\{\begin{array}{c} s_{T}=0.28\;\text{PMV}/{}^{\circ}C\\ s_{RH}=0.007\;\text{PMV}/\%\text{RH} \end{array}\right.$$

Having determined the neutral point $T_{op,0}$ and sensitivity coefficients $s_{T}$ and $s_{RH}$, it was possible to calculate $I_{TC}$ for each participant using Equations (13), (15) and (16). The results show that out of a total of 6404 analyzed points, for all participants and over the entire monitoring period, 10.0% of the data fall in Q4, 12.8% in Q3, 26.5% in Q2, and 50.7% in Q1, with differences between individuals, as shown in Figure 2.

**Figure 5:**
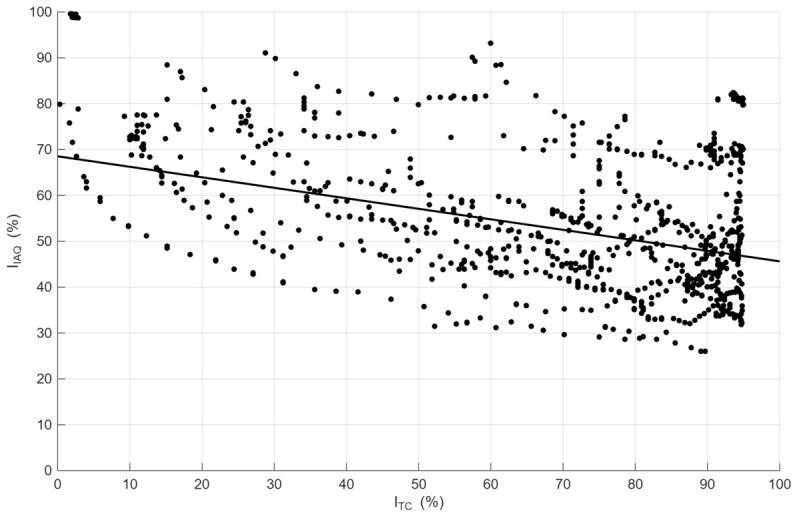
Scatter plot of $I_{IAQ}$ and $I_{TC}$ around the fitted regression line.

**Figure 6:**
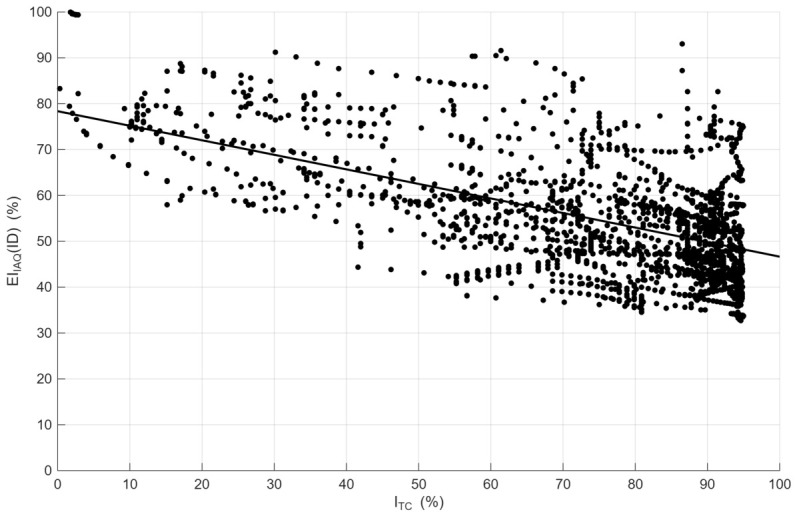
Scatter plot of $EI_{IAQ}\left(ID\right)$ and $I_{TC}$ around the fitted regression line.

The correlation degree between $I_{TC}$ and the other indices is moderate and negative for both $I_{IAQ}$ (R =−0.42, p<0.001) and $EI_{IAQ}\left(ID\right)$ (R = −0.61, p<0.001), indicating that they tend to vary in opposition across the dataset. This trend is confirmed by the observation of a high rate of discrepancies, as shown in Figure 3 for both $I_{IAQ}$ and $EI_{IAQ}\left(ID\right)$. Scatter plots showing the distribution of data points are reported in Figure 5 for $I_{TC}$–$I_{IAQ}$ and Figure 6 for $I_{TC}$–$EI_{IAQ}\left(ID\right)$.

After calculating $I_{IAQ}$, $EI_{IAQ}\left(ID\right)$, and $I_{TC}$ for each participant and each day, the next step was to compute d(ID) using Equation (4). To evaluate the effectiveness of the proposed method in assessing compliance with comfort targets, the results were compared against the state of the art.

For IAQ, we adopted the method proposed by Mujan et al. (2021) [30], which is considered representative of the state of the art [53]. For thermal comfort, ISO 7730 was used, assuming M=1.1 Met, $I_{cl}=1.0$ Clo, and $v_{a}=0.1$ m/s for all occupants. The thresholds were set at 50% for IAQ and 85% for thermal comfort, in accordance with ASHRAE 55 requirements.

To test the proposed method, the comfort thresholds in d(ID) were set as follows:

(19) $$\left\{\begin{array}{c} th_{IAQ}\left(ID\right)=50\\ th_{EIAQ}\left(ID\right)=50\\ th_{TC}\left(ID\right)=80 \end{array}\right.$$

with the same thresholds chosen for all participants. Concerning $th_{TC}\left(ID\right)$, it should ideally be 85% in order to comply with ASHRAE 55; however, considering that $I_{TC}$ was calculated using a worst-case approximation, it is reasonable to add an additional 5% margin. We then proceeded to test the algorithm and count the non-conformity points using the two methods for individual participants and rooms. The results for individual participants are shown in Table 4, while Table 5 reports the non-conformities detected in the monitored rooms.

### 4.2. Interpretation and Discussion of Results

As Figure 2 shows, during this study, neither of the two IAQ indices $I_{IAQ}$ and $EI_{IAQ}$ fell within the quartiles indicating risky conditions. For all participants, the data were distributed within quartiles Q1–Q3, with a high concentration between Q2 and Q3. The correlation between the two indices was strong, specifically, R = 0.72, p<0.001. In fact, both IAQ indices measure different aspects related to the same quantity, i.e., CO_2_. However, the discrepancy condition, defined as the situation in which one index falls in the upper quartiles (Q1–Q2) indicating good conditions while the other falls in Q3–Q4, can be observed for all participants, as reported in Figure 3. This heterogeneity in the data even in the absence of risk conditions shows that $I_{IAQ}$, which is the index most commonly adopted in literature [30], is not sufficient to cover all aspects related to IAQ monitoring, particularly as concerns individual exposure to different pollutant levels. On the other hand, the $EI_{IAQ}\left(ID\right)$ index alone would not be adequate for IAQ monitoring in public buildings, as it is less sensitive to rapid variations compared to $I_{IAQ}$.

With regard to thermal comfort, a moderate negative correlation was observed between $I_{TC}$ and the other indices, $I_{IAQ}$ (R = −0.42, p<0.001) and $EI_{IAQ}\left(ID\right)$ (R = −0.61, p<0.001). This result should be interpreted in the context of the season during which the measurements were taken, i.e., winter. The literature shows that CO_2_ concentration is also an indicator of occupancy levels in indoor environments. In enclosed spaces without external air exchange, temperature tends to rise when people are present, and this increase is greater in more crowded spaces. This intensifies heat exchange between the human body—particularly the warm exhaled air—and the cooler environment. During winter, as observed in the processed results, this can lead to an increase in thermal comfort, which unfortunately corresponds with the deterioration of IAQ due to higher CO_2_ concentrations. However, in summer, when an increase in temperature would reduce thermal comfort, a positive correlation between the indices is expected. This result is consistent with the high rate of discrepancies observed both individually and globally, as shown in Figure 3, referring to the $I_{IAQ}$–$I_{TC}$ and $EI_{IAQ}\left(ID\right)$–$I_{TC}$ pairs. This suggests that no single index alone can provide sufficient information to ensure healthy indoor conditions and that all the components making up the index d(ID) are complementary to one another.

**Figure 7:**
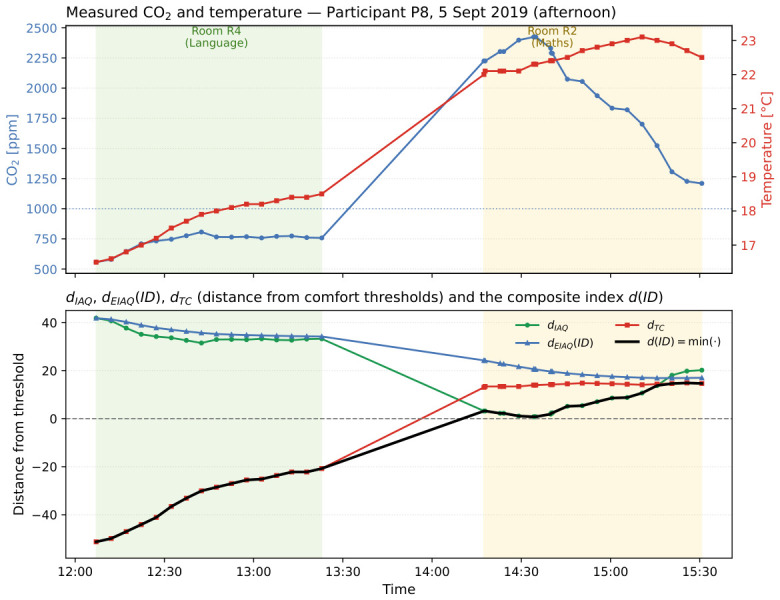
Worked example for participant P8 (5 September 2019, afternoon), showing measured CO_2_ and temperature (**top**) and the resulting component distances and d(ID) (**bottom**).

In this sense, it is useful to illustrate how d(ID) is interpreted through a concrete example drawn from the dataset. Figure 7 reports the measured CO_2_ and temperature for participant P8 during the afternoon of 5 September 2019 together with the resulting component distances $d_{IAQ}$, $d_{EIAQ}\left(ID\right)$, $d_{TC}$, and d(ID)=min(·). During the first period (Room R4, Language class), the room was cool and humid (mean temperature 17.7 °C, mean relative humidity 70%) but well ventilated (mean CO_2_ concentration 730 ppm). This yields $I_{IAQ}=84.3$ and $EI_{IAQ}\left(ID\right)=84.2$, both well above the threshold of 50, but $I_{TC}=47.0$, which is below the threshold of 80. As a result, d(ID)=min(34.3,34.2,−33.0)=−33.0, meaning that the occupant is in a non-compliant condition and that the minimum operator immediately identifies thermal comfort rather than air quality as the responsible factor. A state-of-the-art approach based on $I_{IAQ}$ alone would classify this same period as fully compliant, missing the discomfort condition entirely. During the second period (Room R2, Maths class), the room is warmer and more comfortable (mean temperature 22.5 °C) but less ventilated (mean CO_2_ concentration 2015 ppm), yielding $I_{IAQ}=55.9$, $EI_{IAQ}\left(ID\right)=67.0$, and $I_{TC}=94.5$; here d(ID)=min(5.9,17.0,14.5)=+5.9, a compliant but narrow margin driven by instantaneous air quality. This example illustrates that a negative d(ID) always identifies which of the three components is currently critical, providing building managers with directly actionable information.

Finally, after computing d(ID), the proposed method was tested and validated in comparison to methods from the literature [30,85]. In particular, comfort thresholds were set in order to evaluate the ability of the algorithms to detect one or more of the following conditions: (i) the air quality is medium–low ($I_{IAQ}<50\%$); (ii) the exposure to air quality levels index is medium–low ($EI_{IAQ}<50\%$); and (iii) the thermal comfort index does not comply with the requirements of the ASHRAE 55 standard. As shown in Table 4, the proposed method detects a higher number of non-conformities for all participants compared to the state of the art, demonstrating greater capacity to identify potentially harmful conditions. This result is confirmed when the findings are mapped to the reference rooms actually occupied by the participants during the study. Table 5 reports the non-conformity points detected for each room after eliminating redundancies (i.e., if one or more participants experienced non-conformity levels in a given room at a given time, only one non-conformity point was counted). Even in this case, it can be observed that the proposed method ensures higher safety standards in all rooms.

The impact of sensor measurement uncertainty on both the computed indices and the conformity assessment was evaluated by means of a Monte Carlo simulation. In the simulation, 1000 perturbed datasets were generated by propagating sensor uncertainty through the original measurements, modeled as type-B uncertainty according to manufacturer specifications. This analysis extends the approach adopted in [61] to include the thermal comfort component. The results consistently show that sensor uncertainty produces only limited variations in the indices, with the worst-case deviation always below approximately 5% for all participants and the worst-case variation in the percentage of detected non-conformities below approximately 3% for both methods and below 4% at the room level.

#### Comparison with Existing Approaches

To further clarify the specific contribution of the proposed method with respect to the existing literature, Table 6 compares the proposed solution against representative state-of-the-art and occupant-centered approaches, in terms of four key characteristics: whether the assessment is performed at the individual/occupant level rather than the room level; whether thermal comfort is integrated together with air quality; whether comfort thresholds can be personalized for each occupant; and whether the approach requires wearable devices or dedicated tracking hardware.

As shown in the table, no existing approach combines all four characteristics: room-based methods [30,53] do not provide individual-level assessment; occupant-centric control strategies that integrate thermal comfort and air quality [91] operate at the room level and rely on AI-powered cameras for occupancy detection, rather than on individualized, personalized thresholds; personal monitoring approaches [34,59] achieve individual-level tracking, but require wearable devices or continuous location tracking and do not integrate thermal comfort; finally, the preliminary version of the present framework [61] achieves timetable-based assessment at the individual level without wearable devices, but does not include thermal comfort. Conversely, the present work is able to jointly provide individual-level assessment, thermal comfort integration, personalized thresholds, and freedom from dedicated tracking hardware.

Beyond this qualitative comparison, a quantitative benchmark against the state-of-the-art room-based method in [30] is provided by the non-conformity counts discussed above. When aggregating these counts across all 23 participants, the proposed method detects approximately 14.6% more non-conformity points than the state-of-the-art approach; in the specific case study considered here, this quantifies the additional information gained by jointly considering cumulative exposure and thermal comfort alongside instantaneous air quality.

In terms of computational cost, the evaluation of d(ID) for each occupant at each time step involves only closed-form logarithmic and polynomial expressions (Equations (6)–(16)), with negligible computational cost and no dependency on building size beyond the linear cost of maintaining one record per occupant. Real-time applicability at the scale of the present case study (23 occupants, four rooms) has already been demonstrated through hardware emulation in [61].

### 4.3. Limitations

Several limitations of the present study should be acknowledged. First, the validation dataset included measurements of only CO_2_, temperature, and relative humidity, as discussed in Section 3. The impact of this constraint on the computed indices is conservative by construction, but future validation campaigns should employ multi-pollutant sensor platforms capable of simultaneously measuring TVOC, PM2.5, and CO alongside CO_2_. Second, the monitoring period covered twelve days in September 2019, corresponding to winter in Melbourne, Australia. The negative correlation observed between $I_{TC}$ and the IAQ indices is consistent with winter-specific occupancy dynamics. During summer periods, the relationship is expected to reverse: increased occupancy would reduce thermal comfort while simultaneously worsening IAQ, potentially yielding a positive correlation and a different distribution of non-compliance events. Future studies should include multi-seasonal datasets in order to characterize how the relative contribution of each component to d(ID) varies across seasons. Finally, the thermal comfort model relies on fixed nominal values for metabolic rate, clothing insulation, and air velocity, as justified in Section 2.2.

From a system-integration standpoint, it is worth noting that the min-operator structure underlying $I_{IAQ}$, $EI_{IAQ}\left(ID\right)$, and d(ID) provides graceful degradation in the presence of faulty or missing sensor channels: once a faulty channel is isolated, assessment remains possible using the remaining available measurements, as demonstrated in the CO_2_-only validation of the present case study. A companion implementation study [61] further discusses hardware-level fault tolerance strategies (hardware redundancy and analytical redundancy) for the underlying sensor network.

A further limitation concerns the practical implementation of occupant tracking. The present work infers occupant location from the institutional timetable rather than from real-time positioning technologies such as RFID, Bluetooth Low Energy, WiFi-based localization, or smartphone-based tracking [60]. This choice avoids the cost, deployment complexity, and continuous individual tracking inherent to those technologies, but cannot capture short-term deviations from the schedule such as movement during breaks or unscheduled room changes. In structured environments such as schools and workplaces, where occupancy follows a predefined schedule, these intervals are limited and brief compared to the time spent in scheduled activities, so their impact on cumulative exposure assessment is expected to be small; however, this assumption has not been directly validated against ground-truth positioning data and represents a limitation of the present study.

The use of a unique per-occupant identifier also raises privacy and data protection considerations, particularly given that the target population may include minors. Compliance with data protection regulations such as the General Data Protection Regulation (GDPR) would require, at minimum: a defined legal basis for processing occupancy and exposure data linked to identifiable individuals; data minimization and pseudonymization of the User ID, decoupling it from directly identifying information wherever possible; a Data Protection Impact Assessment given the potential sensitivity of health-related exposure data; and, for minors, parental consent or equivalent safeguards as required by applicable regulations. These aspects are not addressed in the present work, which focuses on the definition and validation of the monitoring metric, and are identified as a necessary component of any real-world deployment.

## 5. Conclusions

This paper has proposed an innovative methodology for optimized monitoring of IAQ in public buildings. In such contexts, occupants often change rooms according to a schedule, being exposed to different air quality conditions during the day. Unlike traditional IAQ monitoring systems, the proposed approach introduces a novel individualized metric that accounts for both environmental comfort levels and personal exposure to pollutants, in comparison to specific thresholds trimmed on personal needs and health conditions of each occupant. The methodology was validated using real-world data from a school case study, showing higher capacity to capture potential harmful or discomfort situations compared to the state of the art. Results also demonstrated that relying solely on a standard IAQ index or thermal comfort index may not be sufficient in situations where room conditions appear to be acceptable but where cumulative personal exposure in fact reveals potential risks. On the other hand, the exposure index alone is not sufficient for IAQ monitoring in public buildings, since it reacts less effectively to rapid changes in air quality. By integrating air quality, exposure over time, and thermal comfort into a unified personal metric, the proposed approach provides a more comprehensive assessment of occupant safety and comfort.

Future perspectives include the implementation of the proposed technology and its validation in real contexts by use of standardized surveys and physiological feedback, which would allow us to assess the impact of the proposed methodology on actual health and comfort. More specifically, future implementations could incorporate wearable sensor data such as skin temperature or activity recognition outputs in order to validate the assumptions made in this paper through occupant-specific estimates of these parameters.

## Figures and Tables

**Table 1:** Regulatory sources used for the computation of α values.

	Regulatory Source
CO_2_	EU Commission Directive 2006/15/EC; ANSI/ASHRAE, 2004
TVOC	FiSIAQ, 2001
PM_2.5_	ANSI/ASHRAE, 2004
CO	WHO 2000; ANSI/ASHRAE, 2004

**Table 2:** Ideal concentration of pollutants for excellent IAQ and α values obtained for the different pollutants [53].

	$C_{X|0}$	$\alpha_{0}$	$\alpha_{1}$	$\alpha_{2}$
CO_2_	415 ppm	63.84	0.02	8.04 × 10^−5^
TVOC	186 ppb	209.60	0.00	0.00
PM2.5	10 µg/m^3^	89.14	0.18	−4.97 × 10^−5^
CO	1.7 ppm	48.58	0.68	−100.36 × 10^−5^

**Table 3:** Technical specifications of the Netamo healthy home coach system.

Monitored Parameter	Units	Range	Accuracy	Resolution
Dry bulb temperature	°C	0–50	±0.3	0.1
Relative Humidity	%	0–100	±3	1
CO_2_	ppm	0–5000	±50 (<1000 ppm)	1
			±5%(≥100 ppm)	
Noise	dB	35–120	N/A	1

**Table 4:** Non-conformities per participant.

Participant	State of the Art	Proposed Method
P1	282	328
P2	227	279
P3	209	230
P4	227	279
P5	230	263
P6	222	261
P7	218	269
P8	87	88
P9	90	101
P10	106	149
P11	109	138
P12	165	185
P13	300	332
P14	232	266
P15	237	266
P16	240	265
P17	237	266
P18	234	257
P19	300	332
P20	237	266
P21	300	332
P22	300	332
P23	162	191

**Table 5:** Non-conformities per room.

Room	State of the Art	Proposed Method
R1	229	282
R2	80	91
R3	252	302
R4	62	63

**Table 6:** Comparison of the proposed method with representative existing approaches. ✓ = characteristic present; × = characteristic absent.

Method	Individual	IAQ	Thermal	Personalized	No Dedicated
	Level		Comfort	Thresholds	Hardware
**Proposed (d(ID))**	✓	✓	✓	✓	✓
Mujan et al. 2021 [30]	×	✓	✓	×	–
De Capua et al. 2023 [53]	×	✓	×	×	–
Wang et al. 2023 [91]	×	✓	✓	×	–
Ruffa et al. 2026 [61]	✓	✓	×	✓	✓
Pan & Covington 2024 [34]	✓	✓	×	×	×
Su et al. 2015 [59]	✓	✓	×	×	×
Jayathissa et al. 2020 [92]	✓	×	✓	×	×
Abdelrahman et al. 2022 [93]	✓	×	✓	×	×
Salamone et al. 2024 [94]	✓	✓	✓	×	×

## Data Availability

No new data were created or analyzed in this study. Data sharing is not applicable to this article.

## Abbreviations

The following abbreviations are used in this manuscript:

ASHRAE	American Society of Heating, Refrigerating, and Air-Conditioning Engineers
DPIA	Data Protection Impact Assessment
GDPR	General Data Protection Regulation
IAQ	Indoor Air Quality
ID	Occupant unique identifier
IoT	Internet of Things
ISO	International Organization for Standardization
PM2.5	Particulate Matter (diameter <2.5μm)
PMV	Predicted Mean Vote
PPD	Predicted Percentage of Dissatisfied
RH	Relative Humidity
TVOC	Total Volatile Organic Compounds
WHO	World Health Organization

## Symbols

The following symbols are used in this manuscript:

α(t)	Time-dependent coefficient in the exposure index
$C_{X}$, $C_{X|0}$	Measured and ideal (baseline) concentration of pollutant *X*
d(ID)	Composite individualized index, $d\left(ID\right)=\min\left(d_{IAQ},d_{EIAQ},d_{TC}\right)$
$d_{IAQ}\left(ID\right),d_{EIAQ}\left(ID\right),d_{TC}\left(ID\right)$	Component distances from comfort thresholds
$EI_{IAQ}\left(ID\right)$	Cumulative exposure-to-air-quality index for occupant ID
$I_{cl}$	Clothing insulation
$I_{IAQ}$	Air quality index of the room
$I_{TC}$	Thermal comfort index, $I_{TC}=100-PPD$
*M*	Metabolic rate
$RH_{0}$	Reference relative humidity (winter neutral point)
$s_{T}$, $s_{RH}$	Worst-case PMV sensitivity to $T_{op}$ and RH
$T_{op}$, $T_{op,0}$	Operative temperature and its winter neutral point
$th_{IAQ}\left(ID\right),th_{EIAQ}\left(ID\right),th_{TC}\left(ID\right)$	Occupant-specific comfort thresholds
$v_{a}$	Air velocity
